# PermaNet Dual, a new deltamethrin-chlorfenapyr mixture net, shows improved efficacy against pyrethroid-resistant *Anopheles gambiae *sensu lato in southern Benin

**DOI:** 10.1038/s41598-023-39140-3

**Published:** 2023-07-28

**Authors:** Thomas Syme, Boris N’dombidjé, Martial Gbegbo, Damien Todjinou, Victoria Ariori, Patricia De Vos, Olivier Pigeon, Corine Ngufor

**Affiliations:** 1 London School of Hygiene & Tropical Medicine, London, UK; 2 Centre de Recherche Entomologique de Cotonou, Cotonou, Benin; 3 Pan African Malaria Vector Research Consortium (PAMVERC), Cotonou, Benin; 4 Centre Walloon de Recherches Agronomiques (CRA-W), Gembloux, Belgium

**Keywords:** Entomology, Infectious diseases

## Abstract

Pyrethroid-chlorfenapyr nets have demonstrated improved entomological and epidemiological impact in trials across Africa. This is driving increased demand for this novel net class in malaria-endemic countries. PermaNet Dual is a new deltamethrin-chlorfenapyr net developed by Vestergaard Sàrl to provide more options to malaria control programmes. We performed an experimental hut trial to evaluate the efficacy of PermaNet Dual against wild, free-flying pyrethroid-resistant *Anopheles gambiae *sensu lato in Covè, Benin. PermaNet Dual induced superior levels of mosquito mortality compared to a pyrethroid-only net and a pyrethroid-piperonyl butoxide net both when unwashed (77% with PermaNet Dual vs. 23% with PermaNet 2.0 and 56% with PermaNet 3.0, *p* < 0.001) and after 20 standardised washes (75% with PermaNet Dual vs. 14% with PermaNet 2.0 and 30% with PermaNet 3.0, *p* < 0.001). Using a provisional non-inferiority margin defined by the World Health Organisation, PermaNet Dual was also non-inferior to a pyrethroid-chlorfenapyr net that has demonstrated improved public health value (Interceptor G2), for vector mortality (79% vs. 76%, OR = 0.878, 95% CIs 0.719–1.073) but not for blood-feeding protection (35% vs. 26%, OR = 1.424, 95% CIs 1.177–1.723). PermaNet Dual presents an additional option of this highly effective net class for improved control of malaria transmitted by pyrethroid-resistant mosquitoes.

## Introduction

Insecticide-treated nets (ITNs) are the most effective and widely adopted preventive measure against malaria. They have been consistently shown to reduce malaria morbidity and mortality under trial^1^ and programmatic conditions^2^, and have made the largest contribution of any intervention to recent reductions in malaria^3^. Their reliance however, on a single insecticide class—the pyrethroids—has exerted selective pressure favouring the spread of pyrethroid resistance in malaria vectors. Between 2010 and 2020, 88% of malaria-endemic countries detected pyrethroid resistance in at least one vector species^4^. Although studies show that ITNs remain protective against malaria infection despite resistance^5^, a substantial body of evidence documents increased survival and blood-feeding of mosquitoes exposed to pyrethroid ITNs^6–9^. Given their importance in malaria prevention and control, any further loss in ITN effectiveness could contribute to resurgences in cases and deaths.

In response to this threat, dual-active ingredient ITNs combining a pyrethroid with another compound designed to restore control of pyrethroid-resistant malaria vectors have been developed. The first novel ITN type combines pyrethroids with piperonyl butoxide (PBO); a synergist that enhances pyrethroid efficacy by neutralising detoxifying enzymes associated with pyrethroid resistance^10^. Pyrethroid-PBO ITNs have shown improved entomological and epidemiological efficacy compared to pyrethroid-only ITNs in experimental hut^11–15^ and cluster-randomised controlled trials (cRCTs)^16,17^. They have since received a conditional recommendation from WHO for distribution in areas where vectors exhibit pyrethroid resistance leading to a significant increase in their deployment in endemic countries in recent years^18^. Pyrethroid-PBO ITNs are not however, without limitations. Notably, there are concerns over their durability following long-term household use^19^. Experimental hut trials in West Africa also suggest that pyrethroid-PBO ITNs may offer more limited benefits in areas with elevated pyrethroid resistance mediated by complex and multiple mechanisms^20^. More ITN types, ideally containing other novel insecticides to which vectors are susceptible are thus needed for effective and sustainable vector control.

More recently, ITNs combining pyrethroids with chlorfenapyr, a pyrrole insecticide that disrupts mitochondrial function, have become available. Chlorfenapyr represents a new mode of action for public health which is suited for the control of vectors that have developed complex mechanisms of resistance to current insecticides. A pyrethroid-chlorfenapyr ITN developed by BASF (Interceptor G2) has been prequalified by WHO^21^, after demonstrating improved control of pyrethroid-resistant malaria vectors in experimental hut trials in Benin^22^, Burkina Faso^23^, Côte d’Ivoire^24^ and Tanzania^25,26^. Evidence of epidemiological impact is also emerging from large-scale trials and pilot distribution schemes in several countries. Most notably, cRCTs in Benin^27^ and Tanzania^28^ showed that Interceptor G2 reduced child malaria incidence by 46% and 44% respectively over 2 years relative to standard pyrethroid-only ITNs. Based on these findings, the WHO has recently released a strong recommendation for the deployment of pyrethroid-chlorfenapyr ITNs over pyrethroid-only ITNs for malaria prevention in areas where vectors are resistant to pyrethroids^29^. This is driving a substantial global increase in demand and order volumes for pyrethroid-chlorfenapyr ITNs for deployment in endemic countries^30^. The development of more innovative varieties of effective pyrethroid-chlorfenapyr nets from multiple manufacturers with robust production capacity, will help improve the health of the ITN market, increasing competition and leading to improved access to more affordable ITN products for optimal vector control impact^31^.

PermaNet Dual is a new deltamethrin-chlorfenapyr ITN developed by Vestergaard Sàrl. Recognising the prohibitive cost and time investment required to conduct cRCTs, to be prequalified by WHO and enter the ITN market successfully, PermaNet Dual must be subjected to semi-field trials to establish its entomological superiority over standard pyrethroid-only ITNs^32,33^. It is also expected to demonstrate non-inferiority to a pyrethroid-chlorfenapyr ITN that has shown empirical evidence of improved public health value. To generate efficacy data as part of a PermaNet Dual dossier submission for assessment by the Prequalification Unit Vector Control Product Assessment Team (PQT/VCP), we performed an experimental hut study to evaluate its efficacy and wash-resistance against wild, free-flying pyrethroid-resistant *Anopheles gambiae *sensu lato (s.l.) in Benin. PermaNet Dual was tested unwashed and after 20 standardised washes and compared to three types of WHO prequalified ITNs; a pyrethroid-only net (PermaNet 2.0), a pyrethroid-PBO net (PermaNet 3.0) and a pyrethroid-chlorfenapyr net (Interceptor G2). Data was analysed to assess the non-inferiority of PermaNet Dual to Interceptor G2 following a recent provisional WHO protocol^32^. The susceptibility of the vector population at the experimental hut site to the insecticides used in the ITNs was assessed during the trial using WHO bottle bioassays. Net pieces cut from ITNs before and after the hut trial were also tested in laboratory bioassays and analysed for chemical content. Following WHO PQT/VCP data requirements, the trial was performed in line with the Organisation for Economic Cooperation and Development (OECD) principles of good laboratory practice (GLP) at the CREC/LSHTM GLP-certified facility in Benin.

## Methods

### Study site

The experimental hut trial was conducted at the CREC/LSHTM field station in Covè, southern Benin (7°14′N2°18′E). The site is located in a vast area of rice irrigation which provides extensive and permanent mosquito breeding sites. *An. coluzzii* and *An. gambiae *sensu stricto (s.s.) occur sympatrically with the former predominating. Recent studies show a high frequency and intensity of pyrethroid and organochlorine resistance but susceptibility to carbamates, organophosphates and pyrroles^34^. Genotyping and gene expression studies have revealed that pyrethroid resistance is mediated by a high frequency of the knockdown resistance (*kdr*) L1014F mutation and overexpression of cytochrome P450 monooxygenases^35^.

### WHO bottle bioassays

WHO bottle bioassays^36^ were performed using F1 progeny of *Anopheles gambiae *s.l. collected as larvae from breeding sites near the experimental hut station to assess the susceptibility of the vector population at the experimental hut station to the active ingredients used in the ITNs. Mosquitoes were exposed to the discriminating concentrations of deltamethrin (12.5 µg) and chlorfenapyr (100 µg). Additional exposures were performed with 2× (25 µg), 5× (62.5 µg) and 10× (125 µg) the discriminating concentration of deltamethrin to determine pyrethroid resistance intensity during the trial. To assess synergism and the contribution of cytochrome P450 monooxygenases to pyrethroid resistance, mosquitoes were also pre-exposed to PBO (400 µg) prior to deltamethrin-coated bottles (12.5 µg). Stock solutions for each insecticide were prepared by dissolving technical grade insecticide in acetone. Test bottles were coated by introducing 1 ml of stock solution into bottles and rotating manually. Approximately 100, unfed, 3–5-day old mosquitoes were exposed to each insecticide and dose for 60 min in four batches of 25. Similar numbers of mosquitoes were concurrently exposed to acetone and PBO-coated bottles as controls. Knockdown was recorded after exposure and mosquitoes were transferred to labelled cups, provided access to 10% (w/v) glucose solution ad libitum and held at 27 ± 2 °C and 75 ± 10% relative humidity (RH). Mortality was recorded after 24 h for deltamethrin and every 24 h up to 72 h for chlorfenapyr.

### Experimental hut trial

Experimental hut trials are standardised simulations of human-occupied housing designed to evaluate the efficacy of indoor vector control interventions against wild, free-flying mosquitoes under controlled field conditions. Host-seeking mosquitoes enter huts at night following attraction by odour cues emanating from human volunteers sleeping inside. Mosquitoes entering the huts then interact freely with the human host and vector control intervention and in the morning, they are collected and scored for physiological and behavioural parameters. The malaria control potential of vector control interventions is assessed primarily in terms of their ability to induce vector mortality (transmission control) and prevent blood-feeding (personal protection).

### Experimental huts

Experimental huts used were of West African design, constructed from concrete bricks with cement-plastered walls, a corrugated iron roof and a polyethylene ceiling. Mosquitoes entered via four window slits with a 1 cm opening positioned on two sides of the hut. A wooden-framed veranda projected from the rear wall of each hut to capture exiting mosquitoes. Huts were surrounded by a water-filled moat to preclude mosquito predators.

### Experimental hut treatments

PermaNet Dual, was compared to three other WHO pre-qualified ITNs; a pyrethroid-only net (PermaNet 2.0), a pyrethroid-PBO net (PermaNet 3.0), and a pyrethroid-chlorfenapyr net (Interceptor G2). A description of the different ITNs tested in the trial is provided below.

- PermaNet Dual (Vestergaard Sàrl) is a candidate 100-denier, polyester ITN coated with a combination of deltamethrin and chlorfenapyr at 2.1 g/kg and 5 g/kg respectively.
- Interceptor G2 (BASF) is a WHO-prequalified 100-denier, polyester ITN coated with a combination of alpha-cypermethrin and chlorfenapyr at 2.4 g/kg and 4.8 g/kg respectively.
- PermaNet 3.0 (Vestergaard Sàrl) is a WHO-prequalified ITN. The roof panel is made of 100-denier, polyethylene monofilament incorporating a combination of deltamethrin and PBO at 4 g/kg and 25 g/kg respectively. The side panels are made of 100-denier, polyester multifilament coated with deltamethrin at 2.1 g/kg.
- PermaNet 2.0 (Vestergaard Sàrl) is a WHO-prequalified polyester ITN coated with deltamethrin at 1.4 g/kg.

An untreated polyester net developed to a similar technical specification as PermaNet® Dual was also tested as a negative control.

All ITNs were tested unwashed and washed 20 times as a proxy for insecticidal loss over 3 years of field use, as per WHO guidelines^37^. Nets were erected in huts by tying the four edges of the roof panel to nails positioned at the upper corners of hut walls. Nets were given 6 holes each measuring 4 × 4 cm to mimic wear-and-tear from routine use. Nine treatments arms were evaluated in nine experimental huts as follows:

1. Untreated polyester net (negative control)
2. PermaNet 2.0—unwashed (deltamethrin only)
3. PermaNet 2.0—washed 20x
4. PermaNet 3.0—unwashed (roof: deltamethrin plus PBO; sides: deltamethrin only)
5. PermaNet 3.0—washed 20x
6. Interceptor G2—unwashed (alpha-cypermethrin plus chlorfenapyr)
7. Interceptor G2—washed 20x
8. PermaNet Dual—unwashed (deltamethrin plus chlorfenapyr)
9. PermaNet Dual—washed 20x

### Experimental hut trial procedure

Human volunteers slept in huts between 21:00 and 06:00 to attract wild, free-flying mosquitoes. Each morning, volunteers collected all mosquitoes from the different compartments of the hut (under the net, room, veranda) using a torch and aspirator and placed them in labelled plastic cups. Mosquito collections were then transferred to the field laboratory for morphological identification and scoring of immediate mortality and blood-feeding. Surviving, female *An. gambiae *s.l. were provided access to 10% glucose (w/v) solution and held at ambient conditions. Delayed mortality was recorded every 24 h up to 72 h to account for the delayed action of chlorfenapyr. Mosquito collections were performed 6 days per week and on the 7th day, huts were cleaned and aired to prevent contamination before the next rotation cycle. Sleepers were rotated between huts daily while treatments were rotated weekly to mitigate the impact of variable host and hut positional attractiveness on mosquito entry. Six replicate nets were also used per treatment and rotated within the treatment daily. The trial continued for one full treatment rotation (9 weeks) between November 2020 and January 2021.

### Experimental hut trial outcome measures

The efficacy of the experimental hut treatments was expressed in terms of the following outcome measures:

1. *Hut entry*—Number of female mosquitoes collected in experimental huts.
2. *Deterrence* (%)—Reduction in the number of mosquitoes collected in the treated hut relative to the untreated control hut. Calculated as follows:
  $$\mathrm{Deterrence }\,\left(\mathrm{\%}\right)= \frac{100(Tu-Tt)}{Tu}$$
  where *Tu* is the number of mosquitoes collected in the untreated control hut and *Tt* is the number of mosquitoes collected in the treated hut.
3. *Exophily* (%)—Exiting rates due to potential irritant effects of a treatment expressed as the proportion of mosquitoes collected in the veranda.
4. *Blood-feeding* (%)—Proportion of blood-fed mosquitoes.
5. *Blood-feeding inhibition* (%)—Proportional reduction in blood-feeding in the treated hut relative to the untreated control hut. Calculated as follows:
  $$\mathrm{Blood \,feeding \,inhibition }\,\left(\mathrm{\%}\right)= \frac{100(Bfu-Bft)}{Bfu}$$
  where *Bfu* is the proportion of blood-fed mosquitoes in the untreated control hut and *Bft* is the proportion of blood-fed mosquitoes in the treated hut.
  *Personal protection* (%)—Reduction in the number of blood-fed mosquitoes in the treated hut relative to the untreated control hut. Calculated as follows:
  $$\mathrm{Personal \,protection }\,\left(\mathrm{\%}\right)= \frac{100(Bu-Bt)}{Bu}$$
  where *Bu* is the number of blood-fed mosquitoes in the untreated control hut and *Bt* is the number of blood-fed mosquitoes in the treated hut.
6. *Delayed mortality* (%)—proportion of dead mosquitoes observed every 24 h up to 72 h after collection.
7. *Overall killing effect* (%)—number of mosquitoes killed in the treated hut relative to the number collected in the untreated control hut. Calculated as follows:
  $$\mathrm{Overall \,killing \,effect}\,\left(\mathrm{\%}\right)= \frac{100(Kt-Ku)}{Tu}$$
  where *Kt* is the number of dead mosquitoes in the treated hut, *Ku* is the number of dead mosquitoes in the untreated control hut and *Tu* is the number of mosquitoes collected in the untreated control hut.

### Preparation of net pieces for bioassays and chemical analysis

For each ITN type, a total of 5 net pieces (one from each panel) measuring 30 × 30 cm were cut before and after the hut trial from randomly selected unwashed and washed nets. Because of the mosaic design of PermaNet 3.0, two additional net pieces were cut from the roof panel to provide 7 pieces in total and a representative sample of pyrethroid-PBO incorporated pieces as per WHO recommendation^38^. Net pieces were wrapped in labelled aluminium foil and stored at 30 °C before and between use for supplementary cone bioassays and tunnel tests. Following use in laboratory bioassays, net pieces were stored at 4 °C before being sent for chemical analysis of insecticide content at the Centre Walloon de Recherches Agronomiques (CRA-W), Belgium.

### Supplementary laboratory bioassays

To provide supplementary data on ITN efficacy, laboratory cone bioassays and tunnel tests were performed with net pieces cut from unwashed and washed ITNs before and after the hut trial. Cone bioassays were performed with the susceptible *An. gambiae *s.s. Kisumu strain to assess the pyrethroid component of ITNs, while tunnel tests were performed with the pyrethroid-resistant *An. gambiae *s.l. Covè strain to assess the chlorfenapyr components of PermaNet Dual and Interceptor G2.

- *An. gambiae *s.s. Kisumu strain is an insecticide-susceptible reference strain originated from Kisumu, western Kenya.
- *An. gambiae *s.l. Covè strain are F1 progeny of mosquitoes collected from the experimental hut site in Covè, southern Benin. It is highly resistant to pyrethroids and organochlorines but susceptible to other insecticide classes including chlorfenapyr. Resistance is mediated by the *kdr* L1014F mutation and overexpression of cytochrome P450 monooxygenases^35^.

All net pieces cut from unwashed and washed ITNs before and after the hut trial were tested in cone bioassays against the susceptible *An. gambiae *s.s. Kisumu strain. Approximately 10, 2–5-day old mosquitoes were exposed to each net piece for 3 min in two replicate cones containing ~ 5 mosquitoes thus giving a total of ~ 50 mosquitoes per treatment arm. After exposure, mosquitoes were transferred to labelled cups, provided access to 10% (w/v) glucose solution and held at 27 ± 2 °C and 75 ± 10% RH. Knockdown was recorded after 60 min and delayed mortality every 24 h up to 72 h.

Previous studies demonstrate the inability of cone bioassays to predict the field efficacy of chlorfenapyr-based ITNs^39^. To assess the efficacy of the chlorfenapyr component of the pyrethroid-chlorfenapyr nets, we therefore performed tunnel tests against the pyrethroid-resistant Covè with two net pieces randomly selected from those cut from unwashed and washed PermaNet 2.0, Interceptor G2 and PermaNet Dual before and after the hut trial. Tunnel tests are an experimental chamber that mimics the behavioural interactions that occur between free-flying mosquitoes and nets during host-seeking. The design consists of a square glass tunnel divided at one third its length by a wooden frame fitted with a net sample. In the short section of the tunnel, a guinea pig bait was held in an open-meshed cage while in the long section, approximately 100, 5–8 day-old mosquitoes were released at dusk and left overnight. Net samples were given 9 holes measuring 1 cm in diameter to facilitate entry of mosquitoes into the baited chamber. In the morning, mosquitoes were collected from the tunnel and scored for mortality and blood-feeding. Surviving mosquitoes were placed in labelled plastic cups, provided access to 10% (w/v) glucose solution, and held at 27 ± 2 °C and 75 ± 10% RH. Delayed mortality was recorded every 24 h up to 72 h. Similar numbers of mosquitoes were concurrently exposed to untreated net pieces in cone bioassays and tunnel tests as a negative control.

### Chemical analysis of net pieces

Following use in bioassays, all net pieces cut from the selected unwashed and washed ITNs before and after the experimental hut trial were assessed for deltamethrin, alpha-cypermethrin, chlorfenapyr and PBO content.

Deltamethrin and/or chlorfenapyr in PermaNet Dual, PermaNet 2.0 and PermaNet 3.0 (sides) were extracted from net samples by sonication with heptane using dicyclohexyl phthalate as internal standard and determined by normal phase High Performance Liquid Chromatography with UV Diode Array Detection (HPLC–DAD). Alpha-cypermethrin and chlorfenapyr in Interceptor G2 were extracted from net samples by sonication with heptane using dicyclohexyl phthalate as internal standard and determined by Gas Chromatography with Flame Ionisation Detection (GC-FID).

Deltamethrin in PermaNet 3.0 (roof) was extracted from net samples by heating under reflux for 30 min with xylene using dicyclohexyl phthalate as internal standard. The solvent was evaporated, and the residue dissolved in hexane. Deltamethrin was determined by normal phase High Performance Liquid Chromatography with UV Diode Array Detection (HPLC–DAD). PBO in PermaNet 3.0 roof was extracted from net samples by heating under reflux for 30 min with xylene using octadecane as internal standard and determined by Gas Chromatography with Flame Ionisation Detection (GC-FID).

Each method of analysis was performed using the internal standard calibration. The analytical methods used were based on validated and standardized methods published by the Collaborative International Pesticides Analytical Council (CIPAC). Chemical analysis results were used to calculate proportional retention of active ingredient(s) and synergist after 20 washes.

### Data analysis

Proportional outcomes (mortality, blood-feeding, exophily) were compared between the experimental hut treatments using blocked logistic regression while numerical outcomes (entry) were compared using negative binomial regression. Separate models were fitted for each outcome and adjusted to account for variation between the huts, sleepers, days of the trial, and wash-point for the pooled analysis. In addition, following recent provisional WHO guidance, PermaNet Dual was assessed for its non-inferiority to Interceptor G2 and its superiority to PermaNet 2.0 and PermaNet 3.0 for mosquito mortality and blood-feeding outcomes. Results with unwashed and washed nets were pooled to generate a single efficacy estimate over the lifetime of the net. All analyses were performed in Stata version 17.

### Ethical considerations

Ethical approval for the study was issued by the Research Ethics Committees of the Benin Ministry of Health (Ref: N°34, 09/09/2020) and the London School of Hygiene & Tropical Medicine (LSHTM) (Ref: 26429). Written informed consent was obtained from all human volunteer sleepers prior to participation. Sleepers were offered a free course of chemoprophylaxis spanning the duration of the study and 4 weeks following its completion to mitigate malaria infection risk. Approval for use of guinea pigs for tunnel tests was obtained from the LSHTM Animal Welfare Ethics Review Board (Ref: 2020-01). Guinea pig colonies were maintained at CREC/LSHTM according to standard operating procedures (SOPs) developed in line with relevant national and international regulations governing use of animals for scientific research purposes. This study is reported in line with Animal Research: Reporting of In Vivo Experiments (ARRIVE) guidelines.

### Compliance with OECD principles of Good Laboratory Practice

To ensure compliance with the OECD principles of GLP, a series of activities were implemented through the initiation, execution, and reporting of the study. The study protocol was developed by a properly trained study director and approved by the sponsor before starting the study. Equipment used for the study (precision balances for weighing insecticides, refrigerators for ITN sample storage and data loggers) were calibrated before use. All ITN products used in the hut trial were verified to be within their expiry dates and were provided with associated certificates of analysis. The candidate net supplied by the manufacturer (Vestergaard Sàrl) was confirmed to come from three production batches. In addition, the environmental conditions under which these products stored were verified daily by use of a calibrated data logger. Mosquitoes used for cone bioassays and tunnel tests were reared and transported in line with established SOPs that ensured the integrity of the strains tested. All computer systems (data loggers, databases, statistical software) used for data collection, entry, and processing, were validated before use. Records were kept of each procedure performed during the study. The quality assurance team of the CREC/LSHTM Facility performed inspections of the study protocol, critical phases of implementation, data quality and final report to assess compliance to GLP and no non-conformances were detected. The final report, along with all study-related documents, are securely stored in the physical and electronic archive of the Facility for up to 15 years. Study inspections performed in 2021 by the South African National Accreditation System (SANAS), the GLP certification body of the Facility, also detected no non-conformances.

## Results

### WHO bottle bioassay results

**Figure 1:**
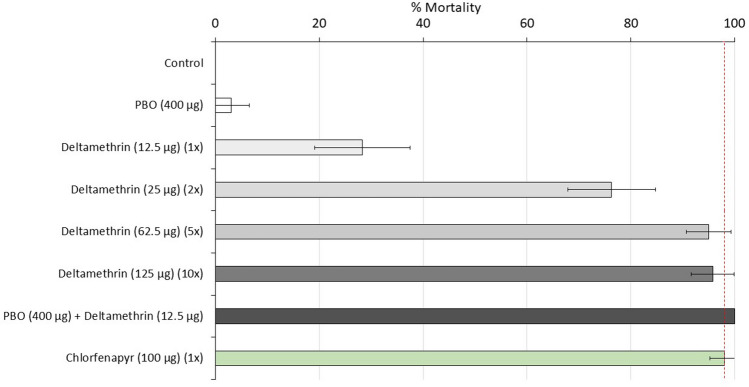
Mortality of F1 progeny of field-collected *Anopheles gambiae *sensu lato in World Health Organisation bottle bioassays. Approximately 100 mosquitoes were exposed to each treatment arm for 60 min in four batches of 25. Dashed red line represents 98% susceptibility cut-off and error bars represent 95% CIs.

Mortality of wild pyrethroid-resistant *An. gambiae *s.l. from the Covè hut station following exposure to the discriminating concentration of deltamethrin was 28% thus confirming the high frequency of pyrethroid resistance in the Covè vector population (Fig. 1). Mortality increased progressively with 2× (76%), 5× (96%) and 10× (96%) the discriminating concentration but failed to exceed 98%, indicating high intensity deltamethrin resistance. Pre-exposure to PBO fully restored deltamethrin susceptibility (100% mortality) thus suggesting the involvement of cytochrome P450 monooxygenases in pyrethroid resistance. In contrast, chlorfenapyr-treated bottles killed 98% of mosquitoes, indicating full susceptibility to this insecticide. No mortality was recorded in the untreated controls while PBO alone induced 3% mortality.

### Experimental hut results

#### Entry and exiting results

A total of 5967 mosquitoes were collected in experimental huts over the 9-week trial, corresponding to an average of approximately 13 mosquitoes per treatment per night (Table 1). None of the ITNs induced a significant deterrent effect relative to the untreated control net and mosquito entry increased significantly with all net types after washing. All ITNs induced significant exiting relative to the control except PermaNet 2.0 after 20 washes (36% vs. 38%, *p* = 0.584). Exiting was higher with all three next-generation ITN types both when unwashed (63–70%) and after 20 washes (56–61%) compared to PermaNet 2.0 (unwashed: 51%, washed: 36%). Mosquito exiting rates did not differ significantly between PermaNet Dual and Interceptor G2 or PermaNet 3.0 both with unwashed nets and nets washed 20 times (*p* > 0.05). Exiting rates declined significantly after washing for all net types except Interceptor G2 (67% vs. 61%, *p* = 0.205).

**Table 1:** Entry and exiting of wild, free-flying, pyrethroid-resistant *Anopheles gambiae *sensu lato entering experimental huts in Covè, southern Benin.

Net type	Net status	Total females caught*	% Deterrence	Total exiting	% Exophily*	95% CIs
Untreated net	–	541^a^	–	208	38.4^a^	34.3–42.5
PermaNet 2.0	Unwashed	490^ab^	9.4	248	50.6^b^	46.2–55.0
	Washed 20x	903^c^	− 66.9	329	36.4^a^	33.3–39.6
PermaNet 3.0	Unwashed	591^bd^	− 9.2	412	69.7^c^	66.0–73.4
	Washed 20x	895^c^	− 65.4	541	60.4^d^	57.2–63.7
Interceptor G2	Unwashed	623^de^	− 15.2	418	67.1^ce^	63.4–70.8
	Washed 20x	669^ cd^	− 23.7	411	61.4^def^	57.7–65.1
PermaNet Dual	Unwashed	459^a^	15.2	291	63.4^cf^	59.0–67.8
	Washed 20x	796^ce^	− 47.1	444	55.8^d^	52.3–59.3

*Values in the same column bearing the same letter do not differ significantly at the 5% level according to regression analysis.

#### Blood-feeding results

**Figure 2:**
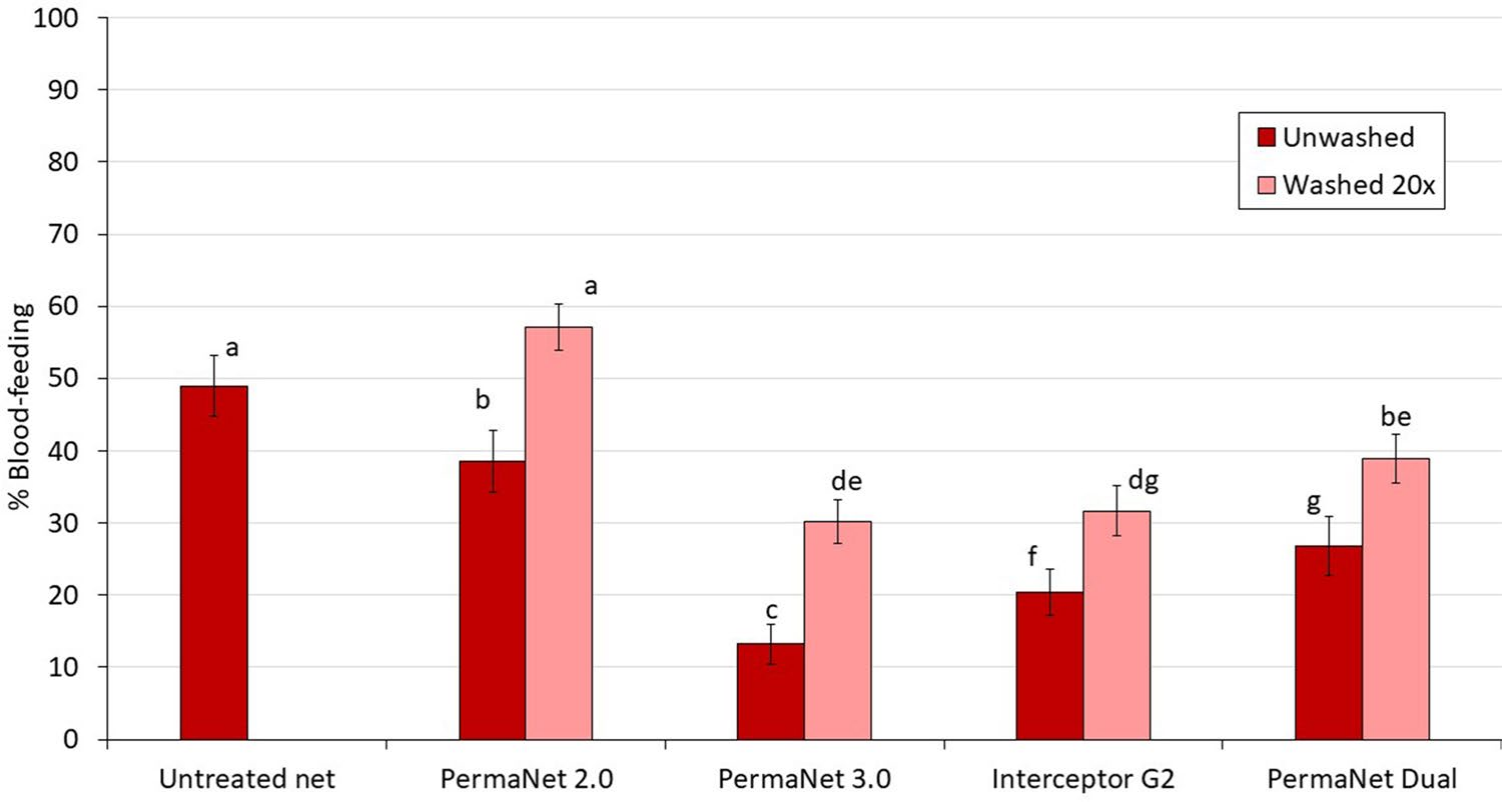
Blood-feeding of wild, free-flying, pyrethroid-resistant *Anopheles gambiae *sensu lato entering experimental huts in Covè, southern Benin. Bars bearing the same letter do not differ significantly at the 5% level according to logistic regression analysis. Error bars represent 95% CIs.

All ITNs significantly reduced blood-feeding relative to the untreated control net except PermaNet 2.0 washed 20 times (57% vs. 49%, *p* = 0.471) (Fig. 2, Table 2). Between unwashed nets, lowest blood-feeding was observed with PermaNet 3.0 when unwashed (13%) though this increased significantly after washing (30%, *p* < 0.001). Interceptor G2 induced lower levels of blood-feeding compared to PermaNet Dual both before washing (20% vs. 27%, *p* = 0.03) and after washing (32% vs. 39%, *p* = 0.006). Personal protection levels were similar between the pyrethroid-chlorfenapyr nets (52% vs. 54%) when unwashed and declined substantially with both net types after 20 washes albeit to a greater extent with PermaNet Dual. Nevertheless, PermaNet Dual provided more blood-feeding inhibition than PermaNet 2.0 before washing (45% vs. 21%) and after 20 washes (21% vs. − 17%). For all ITN-types, blood-feeding rates were significantly higher with washed nets compared to unwashed nets (*p* < 0.05).

**Table 2:** Blood-feeding of wild, free-flying, pyrethroid-resistant *Anopheles gambiae *sensu lato entering experimental huts in Covè, southern Benin.

Net type	Net status	Total females caught*	Total blood-fed	% Blood-feeding*	95% CIs	% Blood-feeding inhibition	% Personal protection
Untreated net	–	541^a^	265	49.0^a^	44.8–53.2	–	–
PermaNet 2.0	Unwashed	490^ab^	189	38.6^b^	34.3–42.9	21.3	28.7
	Washed 20x	903^c^	516	57.1^a^	53.9–60.4	− 16.7	− 94.7
PermaNet 3.0	Unwashed	591^bd^	78	13.2^c^	10.5–15.9	73.1	70.6
	Washed 20x	895^c^	270	30.2^de^	27.2–33.2	38.4	− 1.9
Interceptor G2	Unwashed	623^de^	127	20.4f.	17.2–23.5	58.4	52.1
	Washed 20x	669^ cd^	212	31.7^dg^	28.2–35.2	35.3	20.0
PermaNet Dual	Unwashed	459^a^	123	26.8^ g^	22.7–30.8	45.3	53.6
	Washed 20x	796^ce^	310	38.9^be^	35.6–42.3	20.5	− 17.0

*Values in the same column bearing the same letter do not differ significantly at the 5% level according to logistic regression analysis.

#### Mortality results

**Figure 3:**
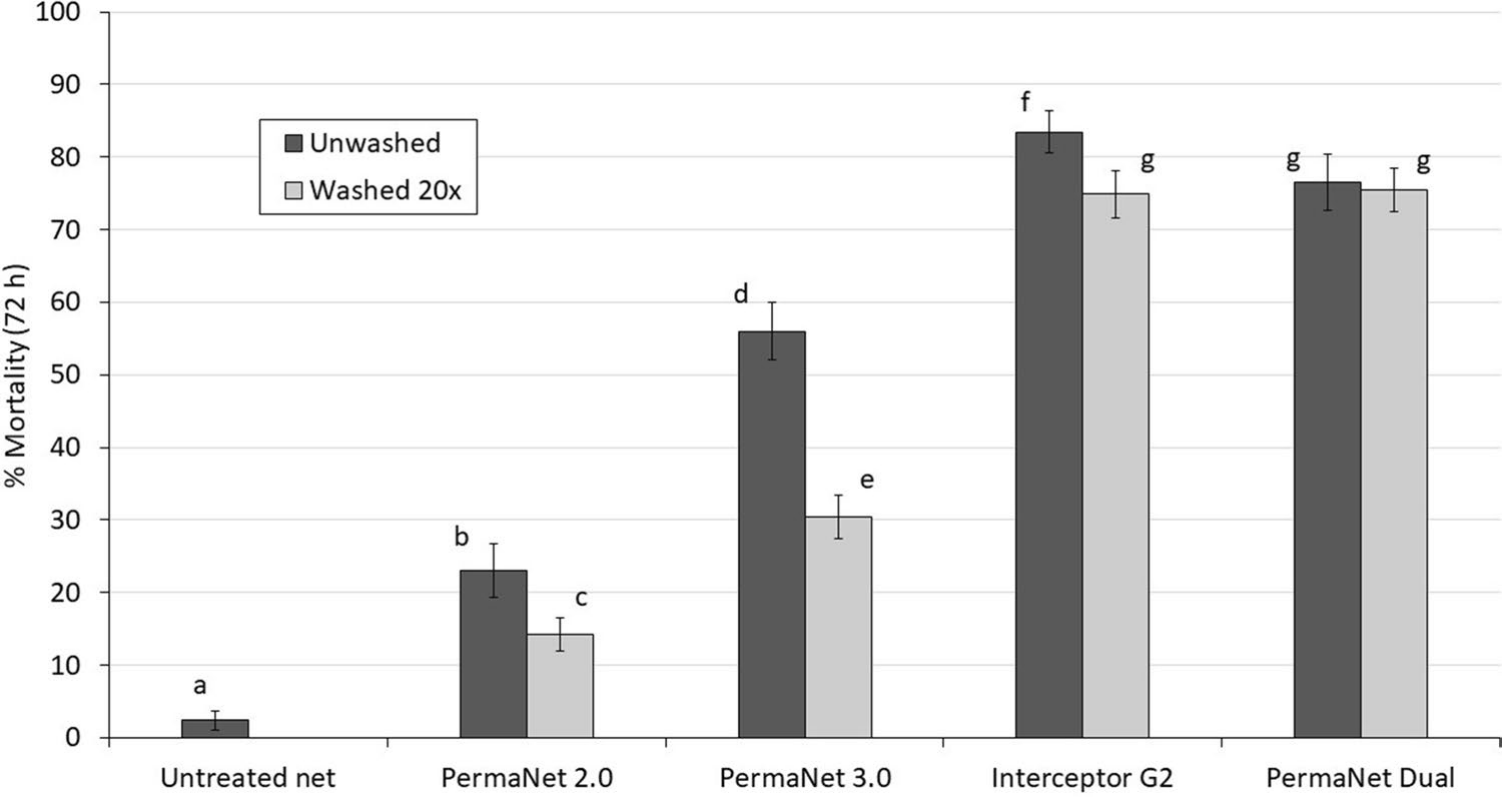
Mortality (72 h) of wild, free-flying, pyrethroid-resistant *Anopheles gambiae *sensu lato entering experimental huts in Covè, southern Benin. Bars bearing the same letter do not differ significantly at the 5% level according to logistic regression analysis. Error bars represent 95% CIs.

Mortality of wild free-flying pyrethroid-resistant *An. gambiae *s.l. with the untreated control net was 2% (Fig. 3, Table 3). Among the ITNs, lowest mosquito mortality was achieved with PermaNet 2.0 (unwashed: 23%, washed: 14%). PermaNet 3.0 induced higher mortality than PermaNet 2.0 both with unwashed nets (56% vs. 23%, *p* < 0.001) and nets washed 20 times (30% vs. 14%, *p* < 0.001). Mortality decreased significantly after washing with both PermaNet 2.0 (23% vs. 14%, *p* = 0.002) and PermaNet 3.0 (56% vs. 30%, *p* < 0.001). The pyrethroid-chlorfenapyr nets induced significantly higher levels of mosquito mortality (76–83% when unwashed and 75% with both net types after 20 washes) compared to PermaNet 2.0 and PermaNet 3.0 (*p* < 0.001). Interceptor G2 induced higher vector mortality than PermaNet Dual when unwashed (83% vs. 76%, *p* = 0.019) but similar mortality after 20 washes (75% vs. 75%, *p* = 0.865). While a significant decline in vector mortality was observed with Interceptor G2 after 20 washes (83% to 75%, *p* = 0.002), the levels of mortality achieved with PermaNet Dual remained similar after washing (76% vs. 75%, *p* = 0.684).

**Table 3:** Mortality of wild, free-flying, pyrethroid-resistant *Anopheles gambiae* sensu lato entering experimental huts in Covè, southern Benin.

Net type	Net status	Total females caught*	Total 72 h mortality	% 72 h Mortality*	95% CIs	% Overall killing effect
Untreated net	**–**	541^a^	13	2.4^a^	1.1–3.7	–
PermaNet 2.0	Unwashed	490^ab^	113	23.1^b^	19.4–26.8	18.5
	Washed 20x	903^c^	128	14.2^c^	11.9–16.5	21.3
PermaNet 3.0	Unwashed	591^bd^	331	56.0^d^	52.0–60	58.8
	Washed 20x	895^c^	272	30.4^e^	27.4–33.4	47.9
Interceptor G2	Unwashed	623^de^	520	83.5f.	80.6–86.4	93.7
	Washed 20x	669^ cd^	501	74.9^ g^	71.6–78.2	90.2
PermaNet Dual	Unwashed	459^a^	351	76.5^ g^	72.6–80.4	62.5
	Washed 20x	796^ce^	600	75.4^ g^	72.4–78.4	108.5

*Values in the same column bearing the same letter do not differ significantly at the 5% level according to logistic regression analysis.

#### Non-inferiority assessment

Following provisional WHO guidelines recommending a non-inferiority margin of 0.7, PermaNet Dual was considered non-inferior to Interceptor G2 for mortality if the lower 95% confidence interval (CI) of the odds ratio describing the difference in mortality was greater than 0.7 and for blood-feeding if the upper 95% CI estimate of the odds ratio describing the difference in blood-feeding was lower than 1.43. PermaNet Dual was also tested for its superiority over PermaNet 2.0 and PermaNet 3.0 for mortality and blood-feeding outcomes. For the non-inferiority and superiority assessments, results with unwashed and washed nets were pooled to generate a single efficacy estimate over the lifetime of the net. As per the recommendations of a recent WHO technical consultation^40^, mortality was adopted as the primary endpoint to assess the non-inferiority of PermaNet Dual while blood-feeding was included as a secondary endpoint to support programmatic decision-making.

**Figure 4:**
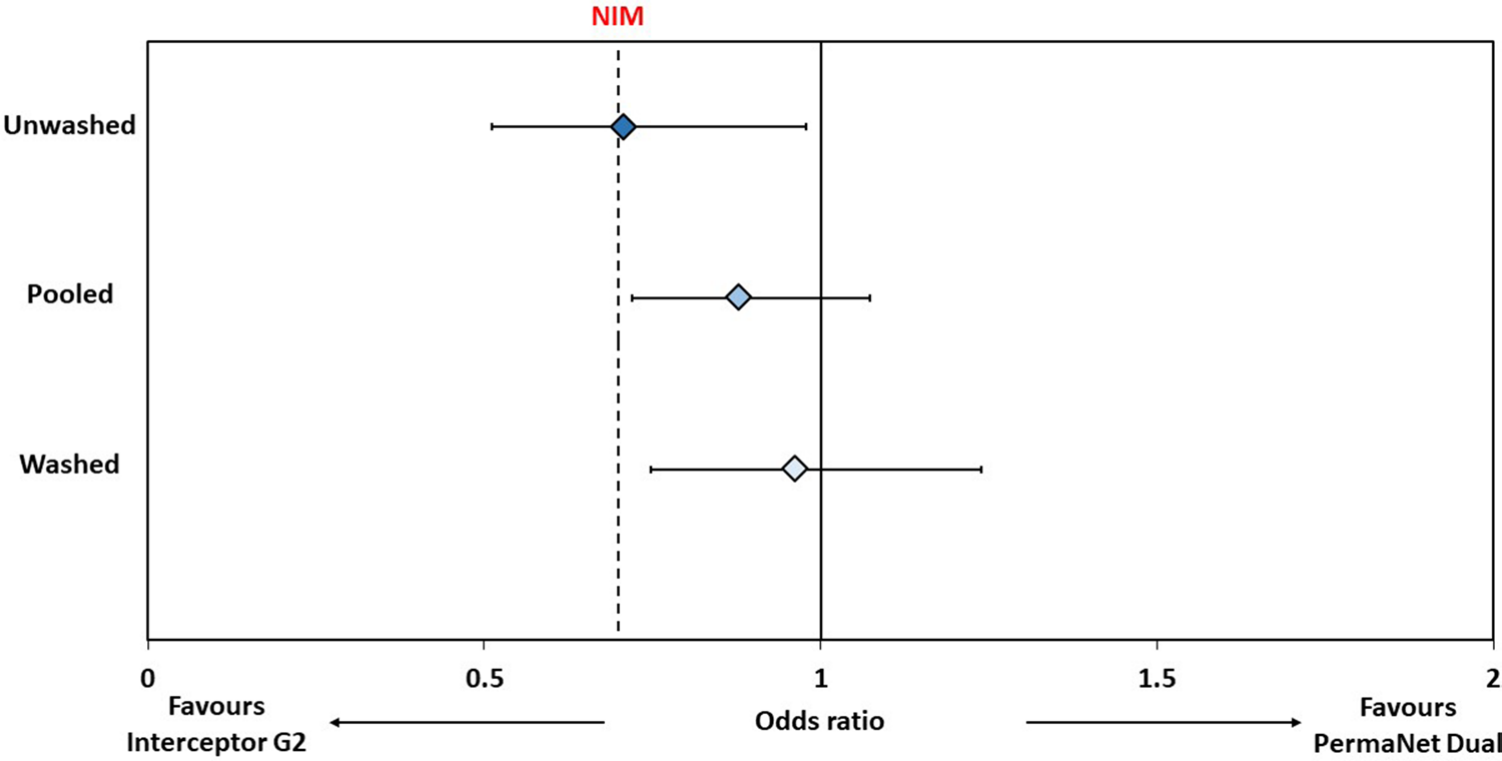
Non-inferiority analysis for proportional mosquito mortality in experimental huts for PermaNet Dual compared to Interceptor G2. Odds ratios represented by blue-shaded diamonds for unwashed nets, washed nets and pooled analysis. Error bars represent 95% CIs. Dashed line represents margin of non-inferiority (odds ratio = 0.7). Lower 95% CI must exceed dashed line to fulfill non-inferiorty criteria.

**Figure 5:**
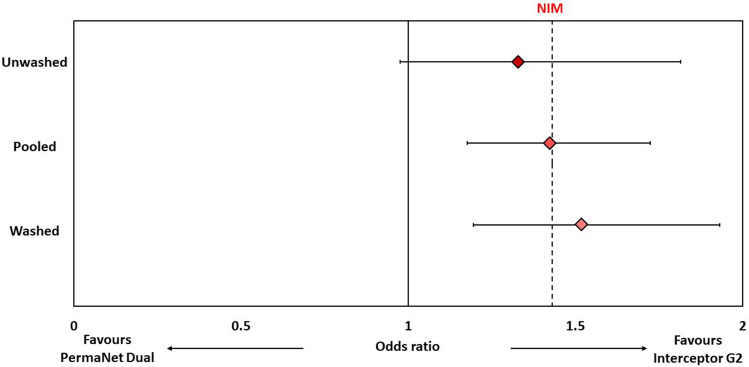
Non-inferiority analysis for proportional mosquito blood-feeding in experimental huts for PermaNet Dual compared to Interceptor G2. Odds ratios represented by red-shaded diamonds for unwashed nets, washed nets and pooled analysis. Error bars represent 95% CIs. Dashed line represents margin of non-inferiority (odds ratio = 1.43). Upper 95% CI must not exceed dashed line to fulfill non-inferiorty criteria.

The odds ratio describing the difference between PermaNet Dual and Interceptor G2 was 0.878 for mortality (76% vs. 79%, 95% CIs 0.719–1.073) and 1.424 for blood-feeding (35% vs. 26%, 95% CIs 1.177–1.723) (Figs. 4 and 5, Table 4). Based on the non-inferiority margin outlined above, PermaNet Dual was therefore non-inferior to Interceptor G2 in terms of its ability to kill vector mosquitoes but not non-inferior in terms of its ability to prevent blood-feeding. PermaNet Dual demonstrated superiority over PermaNet 2.0 both in terms of vector mortality (76% vs. 17%, *p* < 0.001) and blood-feeding (35% vs. 51%, *p* < 0.001). PermaNet Dual was also superior to PermaNet 3.0 in terms of inducing vector mortality (76% vs. 41%, *p* < 0.001) however, it was inferior in terms of preventing blood-feeding (35% vs. 23%, *p* < 0.001). Detailed results from the non-inferiority and superiority assessments are provided in Table S1.

**Table 4:** Non-inferiority analyses comparing the effect of PermaNet Dual to Interceptor G2 for mosquito mortality and blood-feeding outcomes in experimental huts.

	Unwashed		Washed		Pooled	
	Odds ratio (95% CIs)	Non-inferiority	Odds ratio (95% CIs)	Non-inferiority	Odds ratio (95% CIs)	Non-inferiority
Mortality	0.708 (0.512–0.978)	Not non-inferior	0.962 (0.747–1.238)	Non-inferior	0.878(0.719–1.073)	Non-inferior
Blood-feeding	1.330 (0.976–1.814)	Not non-inferior	1.519 (1.195–1.932)	Not non-inferior	1.424 (1.177–1.723)	Not non-inferior

To fulfill non-inferiority criteria, lower 95% CI of odds ratio must exceed 0.7 for mortality while upper 95% CI of odds ratio must not exceed 1.43 for blood-feeding.

#### Supplementary laboratory bioassay results

**Figure 6:**
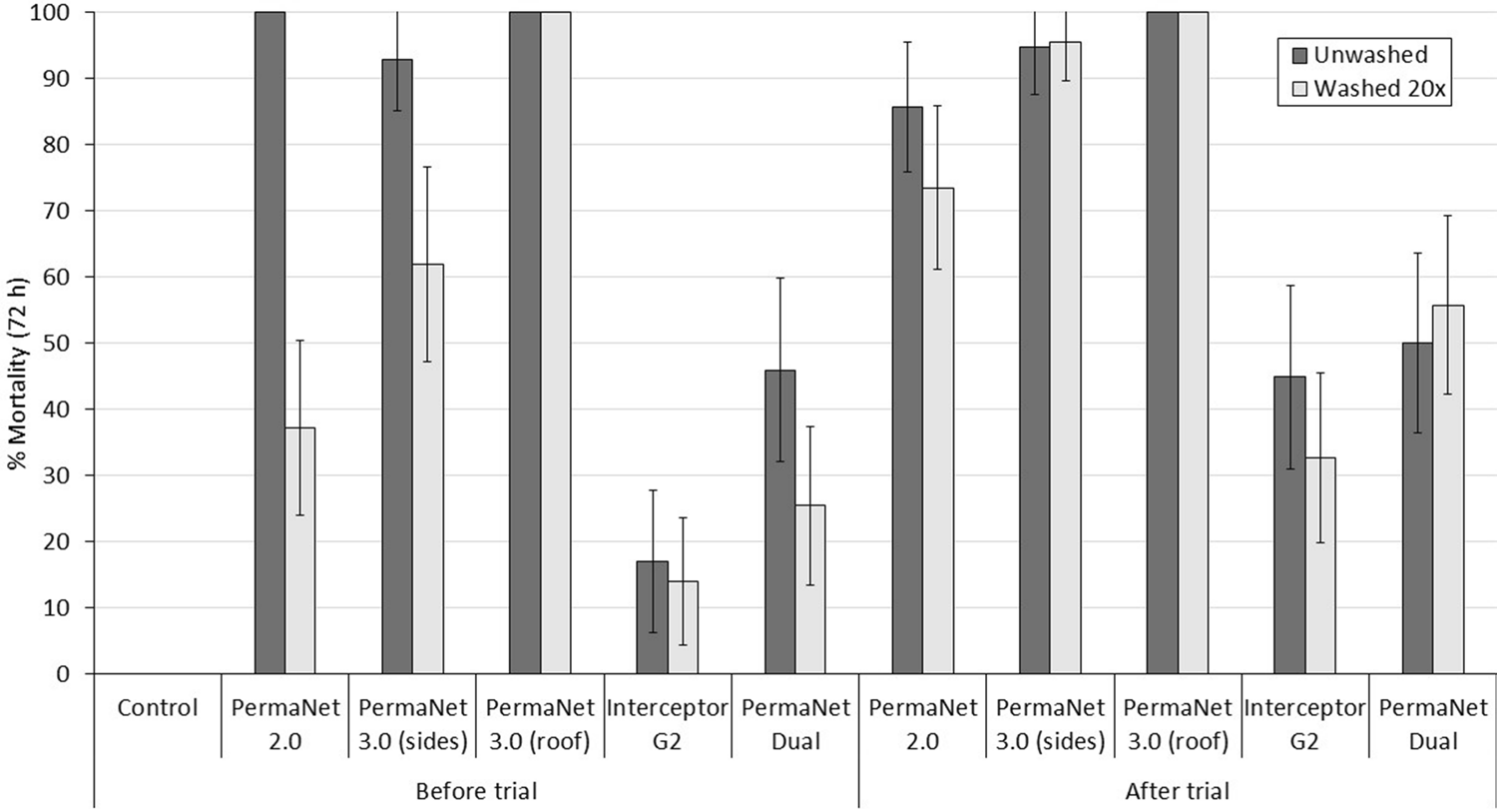
Mortality after 72 h of susceptible *Anopheles gambiae *sensu stricto Kisumu strain in supplementary cone bioassays. Approximately 10 mosquitoes were exposed to each of the 5 net pieces cut from unwashed and washed nets before and after the hut trial for 3 min in two batches of 5. Error bars represent 95% CIs.

**Figure 7:**
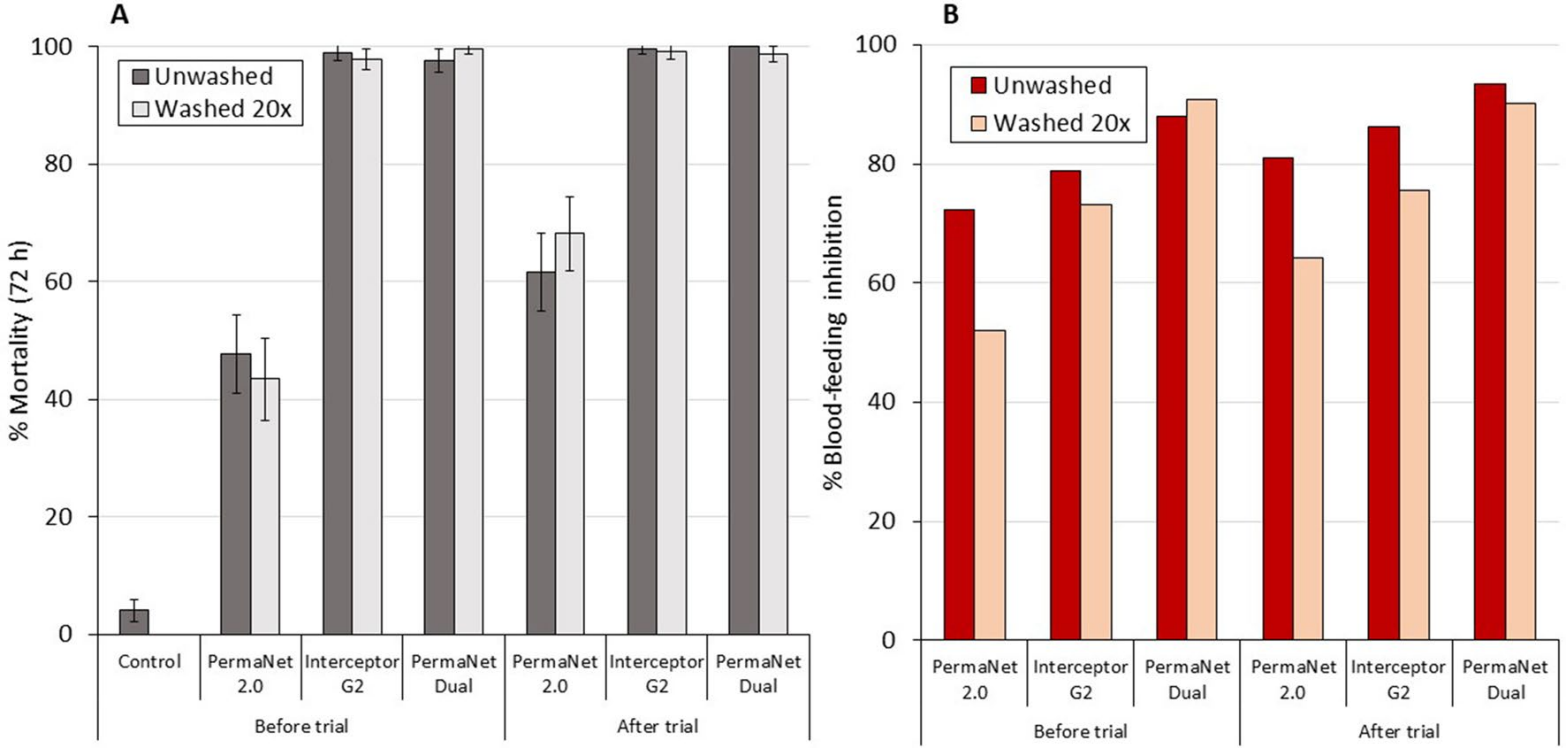
Mortality after 72 h (**A**) and blood-feeding inhibition (**B**) of pyrethroid-resistant *Anopheles gambiae *sensu lato Covè strain in supplementary tunnel tests. Approximately 100 mosquitoes were exposed to each of the two randomly selected net pieces from each treatment arm overnight in one replicate tunnel test. Error bars represent 95% CIs.

Cone bioassay results with the susceptible *An. gambiae *s.s. Kisumu strain and tunnel test results with the *An. gambiae *s.l. Covè strain are provided in Figs. 6 and 7 respectively with more detailed results provided in supplementary information (Tables S2 and S3). PermaNet 3.0 roof samples induced the highest mortality rates in cone bioassays (78–97%). As expected, the performance of the pyrethroid-chlorfenapyr nets was very poor in cone bioassays inducing < 60% mortality with all ITN pieces tested. Hence, the results reaffirm the unsuitability of cone bioassays for testing pyrethroid-chlorfenapyr ITNs.

Tunnel test mortality with the pyrethroid-resistant Covè strain was lowest with PermaNet 2.0, (< 70%) though this did not decline significantly after washing (Fig. 6). In contrast, unwashed and washed net pieces of both pyrethroid-chlorfenapyr ITNs taken before and after the hut trial induced ≥ 98% mortality. Blood-feeding inhibition was high with all ITNs (50–93%). Highest blood-feeding inhibition was achieved with PermaNet Dual, exceeding 85% with all net pieces and was similar between unwashed and washed pieces taken before (88% vs. 91%) and after the hut trial (93% vs. 90%).

#### Chemical analysis of net pieces results

The active ingredient content in all unwashed ITNs was within defined specifications declared by the manufacturers. Retention of deltamethrin after 20 washes was lowest with net pieces cut from PermaNet 2.0 (17%) (Table 5). PermaNet 3.0 showed higher proportional wash-retention of deltamethrin on the roof panel (86%) compared to the side panels (26%). The PBO component showed moderate levels of wash-retention (60%). Between the pyrethroid–chlorfenapyr ITNs, Interceptor G2 showed higher levels of wash-retention of both active ingredients (87% for alpha-cypermethrin and 65% for chlorfenapyr) compared to PermaNet Dual (42% for deltamethrin and 25% for chlorfenapyr). The wash-resistance index of chlorfenapyr was thus higher with Interceptor G2 (98%) than with PermaNet Dual (93%).

**Table 5:** Chemical content of unwashed and washed net pieces taken before and after the experimental hut trial in Covè, Benin.

ITN type	Active ingredient(s)	AI content (g/kg)		AI retention (%)
		Unwashed	Washed 20x	
PermaNet 2.0	Deltamethrin	1.25	0.21	16.8
PermaNet 3.0	Deltamethrin (sides)	2.09	0.54	25.8
	Deltamethrin (roof)	4.03	3.47	86.1
	PBO (roof)	23.2	13.9	59.9
Interceptor G2	Alpha-cypermethrin	2.42	2.1	86.8
	Chlorfenapyr	5.52	3.6	65.2
PermaNet Dual	Deltamethrin	2.29	0.96	41.9
	Chlorfenapyr	5.17	1.29	25.0

## Discussion

This study evaluated the efficacy and wash resistance of PermaNet Dual—a new deltamethrin-chlorfenapyr net—against a pyrethroid-resistant malaria vector population in an experimental hut trial in southern Benin. PermaNet Dual was investigated for its superiority to WHO-prequalified pyrethroid-only (PermaNet 2.0) and pyrethroid-PBO (PermaNet 3.0) ITNs and non-inferiority to a WHO-prequalified pyrethroid-chlorfenapyr ITN (Interceptor G2) with empirical evidence of public health value.

The poor performance of PermaNet 2.0 (< 25% mortality) is typical of experimental hut trials conducted with pyrethroid-only nets at the Covè hut site and is attributable to the high intensity of pyrethroid resistance demonstrated in susceptibility bottle bioassays in this study and in previous^34,35^. Complete restoration of susceptibility to deltamethrin following pre-exposure to PBO was observed in the bottle bioassays which suggests strong involvement of cytochrome P450 monooxygenase activity in deltamethrin resistance in the Covè vector population during the hut trial. Previous bioassays performed with wild *An. gambiae *s.l. from Covè using different pyrethroid insecticides have often resulted in partial or no restoration of susceptibility to pyrethroids with pre-exposure to PBO. This variability in outcome of synergist bioassays may be due to differences in the type of pyrethroid insecticide tested, test methods or seasonal changes in the vector population. However, despite complete restoration of pyrethroid susceptibility following PBO pre-exposure in bottle bioassays in this hut trial, the levels of improved mosquito mortality achieved with the PermaNet 3.0 relative to PermaNet 2.0 were moderate (17% vs. 40%) and did not differ substantially compared to what has been reported in previous hut studies with this vector population^20,41^. This finding would suggest the presence of more complex behavioural mechanisms that may have reduced mosquito contact with PermaNet 3.0 compromising its efficacy in the experimental huts. Further studies to investigate the relationship between levels of restoration of susceptibility to pyrethroids achieved in PBO pre-exposure bioassays and the efficacy of pyrethroid-PBO ITNs would be useful.

Both pyrethroid-chlorfenapyr ITNs (PermaNet Dual and Interceptor G2) induced significantly higher levels of mortality (75–86%) of wild pyrethroid-resistant malaria vector mosquitoes entering the experimental huts relative to the pyrethroid-only and pyrethroid-PBO ITNs (PermaNet 2.0 and PermaNet 3.0). Similar findings were obtained in parallel experimental hut trials with PermaNet Dual in Côte d’Ivoire^42^. This superior performance is mostly due to susceptibility of the local vector population to chlorfenapyr as demonstrated in bottle bioassays performed with wild vector mosquitoes from the Covè hut station during the trial. The results confirm previous findings with pyrethroid-chlorfenapyr ITNs in experimental hut studies in Benin^22,43^ and across Africa^23–25^ and recent cRCTs in Benin^27^ Tanzania^28^, reiterating the importance of this innovative ITN technology for improving the control of pyrethroid-resistant malaria vector populations. PermaNet Dual was also non-inferior to Interceptor G2 for the primary endpoint of mortality thus providing necessary evidence for the candidate ITN to be covered by recent WHO policy recommendations for deployment of pyrethroid-chlorfenapyr nets^29^. Studies investigating its entomological performance against other malaria vector species in other ecological settings are ongoing and will add to the body of evidence to support its deployment. The recent prequalification of PermaNet Dual by WHO^44^ therefore provides an additional choice of effective pyrethroid-chlorfenapyr nets to vector control programmes and gives opportunity for procurers to meet the increasing global demand for this effective dual-active ITN class by endemic countries.

Experimental hut performance after 20 washes is used as a proxy for ITN efficacy after 3 years of field use^37^. Although wash-retention of chlorfenapyr was lower in PermaNet Dual relative to Interceptor G2, its performance in experimental huts remained unchanged after 20 standardised washes, showing potential for the net to demonstrate durable bioefficacy. This finding was supported by the tunnel tests demonstrating high mortality of pyrethroid-resistant Covè mosquitoes (> 95%) with the two pyrethroid-chlorfenapyr ITNs, both before and after 20 washes. However, further studies to monitor the post-market performance of PermaNet Dual including assessment of its fabric integrity, bioefficacy and chemical content under household use over 3 years, are advisable.

While a superior performance of pyrethroid-chlorfenapyr nets relative to pyrethroid-only and pyrethroid-PBO nets was clearly demonstrated in this study and in previous hut studies and cRCTs, care should be taken not to over-rely on this one class of insecticide as this may quickly drive development of resistance to chlorfenapyr eventually leading to product failure. Pyrethroid-chlorfenapyr nets should be ideally deployed alongside other insecticide chemistries or in rotation with other ITN types as part of an insecticide resistance management strategy aimed at preventing the selection of chlorfenapyr resistance and extending the useful life of this ITN class.

## Conclusions

PermaNet Dual, a new deltamethrin-chlorfenapyr ITN developed by Vestergaard Sàrl, demonstrated superior performance compared to a pyrethroid-only ITN (PermaNet 2.0) and a pyrethroid-PBO (PermaNet 3.0) ITN in experimental huts against wild, free-flying pyrethroid-resistant *An gambiae *s.l. in Benin. PermaNet Dual was also non-inferior to Interceptor G2, a WHO-prequalified pyrethroid-chlorfenapyr ITN that has demonstrated evidence of improved public health impact in cRCTs. The addition of PermaNet Dual to the current WHO list of prequalified ITNs presents an additional option of this highly effective ITN class for improved control of malaria transmitted by pyrethroid-resistant mosquito vectors.

## Supplementary Information

Supplementary Tables.

## Data Availability

The datasets used and/or analysed during the current study are available from the corresponding author on reasonable request.

## Abbreviations

PBO: Piperonyl butoxide
ITN: Insecticide treated nets
LLIN: Long-lasting insecticidal nets
WHO: World Health Organization
PQ: Prequalification team
cRCT: Cluster randomised controlled trial
GLP: Good laboratory practice
CREC: Centre de Recherche Entomologique de Cotonou
LSHTM: London School of Hygiene & Tropical Medicine
