# Supplementary material for: PermaNet Dual, a new deltamethrin-chlorfenapyr mixture net, shows improved efficacy against pyrethroid-resistant Anopheles gambiae sensu lato in southern Benin
Source: Sci Rep. 2023 Jul 28;13:12232. doi: 10.1038/s41598-023-39140-3 (PMC10382523; doi:10.1038/s41598-023-39140-3)
Supplement: Supplementary file 1 — Supplementary Tables. [file 41598_2023_39140_MOESM1_ESM.pdf]

Supplementary information

PermaNet Dual, a new deltamethrin-chlorfenapyr mixture net, shows improved efficacy against pyrethroid-resistant *Anopheles gambiae sensu lato* in southern Benin

Thomas Syme[1,2,3*], Boris N'dombidjé[2,3], Martial Gbegbo[2,3], Damien Todjinou[2,3], Victoria Ariori[2,3], Patricia De Vos[4], Olivier Pigeon[4], Corine Ngufor[1,2,3*]

[1]London School of Hygiene & Tropical Medicine, United Kingdom
[2]Centre de Recherche Entomologique de Cotonou, Benin
[3]Pan African Malaria Vector Research Consortium (PAMVERC), Benin
[4]Centre Walloon de Recherches Agronomiques (CRA-W), Belgium

*Corresponding authors

(CN) Email: corine.ngufor@lshtm.ac.uk

(TS) Email: Thomas.syme@lshtm.ac.uk

Table S1: Non-inferiority and superiority analyses comparing the effect of PermaNet Dual to Interceptor G2, PermaNet 2.0 and PermaNet 3.0 for mosquito mortality and blood-feeding outcomes in experimental huts.

		Superiority assessments				Non-inferiority assessment	
		**PermaNet 2.0**	**PermaNet Dual**	**PermaNet 3.0**	**PermaNet Dual**	**Interceptor G2**	**PermaNet Dual**
	**Total collected**	1393	1255	1486	1255	1292	1255
**Mortality (primary endpoint)**	**Total dead**	241	951	599	951	1021	951
	**Mortality (%)**	17.3	75.8	40.3	75.8	79.0	75.8
	**Odds ratio**	–	18.012	–	5.564	–	0.878
	**Std. error (on log odds scale)**	–	1.900	–	0.531	–	0.089
	**P-value**	–	<0.001	–	<0.001	–	0.205
	**95% CIs**	–	14.645–22.148	–	4.614–6.709	–	0.719–1.073
	**WHO efficacy criteria**	–	Significantly higher (p<0.05)	–	Significantly higher (p<0.05)	–	Lower 95% CI >0.7
	**Conclusion**	–	**Superior**	–	**Superior**	–	**Non-inferior**
**Blood-feeding (secondary endpoint)**	**Total blood-fed**	705	433	348	433	339	433
	**Blood-feeding (%)**	50.6	34.5	23.4	34.5	26.2	34.5
	**Odds ratio**	–	0.495	–	1.578	–	1.424
	**Std. error (on log odds scale)**	–	0.044	–	0.151	–	0.138
	**P-value**	–	<0.001	–	<0.001	–	<0.001
	**95% CIs**	–	0.415–0.590	–	1.308–1.903	–	1.177–1.723
	**WHO efficacy criteria**	–	Significantly lower (p<0.05)	–	Significantly lower (p<0.05)	–	Upper 95% CI <1.43
	**Conclusion**	–	**Superior**	–	**Inferior**	–	**Not non-inferior**

Table S2: Supplementary cone bioassay results with the susceptible *Anopheles gambiae sensu stricto* Kisumu strain. *A total of 40–60 mosquitoes were exposed to each of the five net pieces per treatment arm for 3 mins in ten batches of 4–6.*

**Net type**	**Net status**	**N**	**N KD**	**% KD**	**95% CIs**	**N dead 24 h**	**% dead 24 h**	**N dead 48 h**	**% dead 48 h**	**N dead 72 h**	**% dead 72 h**	**95% CIs**
Control	–	106	0	0	0-5	0	0	0	0	0	0	0-5.0
PermaNet 2.0	Unwashed before trial	55	55	100	95.0-100	53	96.4	55	100	55	100	95.0-100
	Washed 20x before trial	51	39	76.5	64.9-88.1	13	25.5	14	27.5	19	37.3	24.0-50.6
	Unwashed after trial	49	41	83.7	73.4-94.0	41	83.7	41	83.7	42	85.7	75.9-95.5
	Washed 20x after trial	49	43	87.8	78.6-97.0	33	67.3	35	71.4	36	73.5	61.1-85.9
PermaNet 3.0 (sides)	Unwashed before trial	42	39	92.9	85.1-100	38	90.5	38	90.5	39	92.9	85.1-100
	Washed 20x before trial	42	39	92.9	85.1-100	21	50.0	24	57.1	26	61.9	47.2-76.6
	Unwashed after trial	38	36	94.7	87.6-100	35	92.1	35	92.1	36	94.7	87.6-100
	Washed 20x after trial	45	38	84.4	73.8-95.0	40	88.9	42	93.3	43	95.6	89.6-100
PermaNet 3.0 (roof)	Unwashed before trial	31	27	87.1	75.3-98.9	31	100	31	100	31	100	95.0-100
	Washed 20x before trial	30	30	100	95.0-100	30	100	30	100	30	100	95.0-100
	Unwashed after trial	28	27	96.4	89.5-100	28	100	28	100	28	100	95.0-100
	Washed 20x after trial	31	30	96.8	90.6-100	31	100	31	100	31	100	95.0-100
Interceptor G2	Unwashed before trial	47	36	76.6	64.5-88.7	6	12.8	8	17.0	8	17.0	6.3-27.7
	Washed 20x before trial	50	17	34	20.9-47.1	4	8.0	4	8.0	7	14.0	4.4-23.6
	Unwashed after trial	49	32	65.3	52.0-78.6	20	40.8	21	42.9	22	44.9	31.0-58.8
	Washed 20x after trial	52	34	65.4	52.5-78.3	13	25.0	17	32.7	17	32.7	20.0-45.5
PermaNet Dual	Unwashed before trial	50	45	90	81.7-98.3	22	44.0	22	44.0	23	46.0	32.2-59.8
	Washed 20x before trial	51	27	52.9	39.2-66.6	8	15.7	10	19.6	13	25.5	13.5-37.5
	Unwashed after trial	52	37	71.2	58.9-83.5	24	46.2	24	46.2	26	50.0	36.4-63.6
	Washed 20x after trial	52	32	61.5	48.3-74.7	21	40.4	26	50.0	29	55.8	42.3-69.3

Table S3: Summary tunnel test results with the pyrethroid-resistant *Anopheles gambiae sensu lato* Covè strain. *A total of 160–240 mosquitoes were exposed overnight to each of two randomly selected net pieces per treatment arm in one replicate tunnel test.*

Treatment	Wash status	N exposed	Total Fed	N dead imm	N dead 24 h	N dead 48 h	N dead 72 h	N pass	% Imm Mort	% 24 h mort	% 48 h mort	% 72 h mort	95% CI	% Pass	95% CI	% Bfd	95% CI
Control	N/A	411	299	9	0	3	8	191	2.2	2.2	2.9	4.1	2.2-6.1	46.5	41.7-51.3	72.8	68.5-77.1
PermaNet 2.0	Unwashed before trial	218	44	29	21	38	75	73	13.3	22.9	30.7	47.7	41.1-54.3	33.5	27.2-39.8	20.2	14.9-25.5
	Washed 20 x before trial	198	69	35	17	30	51	65	17.7	26.3	32.8	43.4	36.5-50.3	32.8	26.3-39.4	34.9	28.2-41.5
	Unwashed after trial	211	29	78	25	39	52	70	37.0	48.8	55.5	61.6	55.1-68.2	33.2	26.8-39.5	13.7	9.1-18.4
	Washed 20x after trial	211	55	81	45	53	63	99	38.4	59.7	63.5	68.3	62.0-74.5	46.9	40.2-53.7	26.1	20.2-32.0
Interceptor G2	Unwashed before trial	202	31	187	13	13	13	42	92.6	99.0	99.0	99.0	97.6-100	20.8	15.2-26.4	15.4	10.4-20.3
	Washed 20 x before trial	235	46	212	15	17	18	57	90.2	96.6	97.5	97.9	96.0-99.7	24.3	18.8-29.7	19.6	14.5-24.6
	Unwashed after trial	231	23	223	7	7	7	74	96.5	99.6	99.6	99.6	98.7-100	32.0	26.0-38.1	10.0	6.1-13.8
	Washed 20x after trial	224	40	220	2	2	2	66	98.2	99.1	99.1	99.1	97.9-100	29.5	23.5-35.4	17.9	12.8-22.9
PermaNet Dual	Unwashed before trial	216	19	188	23	23	23	66	87.0	97.7	97.7	97.7	95.7-99.7	30.6	24.4-36.7	8.8	5.0-12.6
	Washed 20 x before trial	224	15	215	8	8	8	53	96.0	99.6	99.6	99.6	98.7-100	23.7	18.1-29.2	6.7	3.4-10.0
	Unwashed after trial	229	11	227	2	2	2	47	99.1	100	100	100	–	20.5	15.3-25.8	4.8	2.0-7.56
	Washed 20x after trial	238	17	229	6	6	6	69	96.2	98.7	98.7	98.7	97.3-100	29.0	23.2-34.8	7.1	3.9-10.4
